# RpfC regulates the expression of the key regulator *hrpX* of the *hrp*/T3SS system in *Xanthomonas campestris* pv. *campestris*

**DOI:** 10.1186/s12866-018-1233-5

**Published:** 2018-09-03

**Authors:** Bo-Le Jiang, Guo-Feng Jiang, Wei Liu, Li-Chao Yang, Li-Yan Yang, Lin Wang, Xiao-Hong Hang, Ji-Liang Tang

**Affiliations:** State Key Laboratory for Conservation and Utilization of Subtropical Agro-bioresources, College of Life Science and Technology, Guangxi University, 100 Daxue Road, Nanning, 530004 Guangxi China

**Keywords:** *Xanthomonas*, RpfC, *hrpX*

## Abstract

**Background:**

The Gram-negative phytopathogenic bacterium *Xanthomonas campestris* pv. *campestris* recruits the *hrp*/T3SS system to inject pathogenicity effector proteins into host cells and uses the *rpf*/DSF cell-cell signaling system to regulate the expression of virulence factors such as extracellular enzymes and polysaccharide. Whether these two systems have any connection is unknown.

**Methods:**

Positive regulator candidates affecting *hrpX* expression were identified by *sacB* strategy. The transcriptional expression was determined by qRT-PCR and GUS activity analysis. Transcriptome analysis was performed by RNA deep-sequencing. The hypersensitive response (HR) was determined in the nonhost plant pepper ECW-10R and electrolyte leakage assay.

**Results:**

Mutation of the gene encoding the sensor RpfC of the *rpf*/DSF system significantly reduced the expression of *hrpX*, the key regulator of the *hrp*/T3SS system, all of the genes in the *hrp* cluster and most reported type III effector genes. Mutation of *rpfG* did not affect the expression of *hrpX*. The *rpfC* mutant showed a delayed and weakened HR induction.

**Conclusions:**

RpfC positively regulates the expression of *hrpX* independent of RpfG, showing a complex regulatory network linking the *rpf/*DSF and *hrp*/T3SS systems.

**Electronic supplementary material:**

The online version of this article (10.1186/s12866-018-1233-5) contains supplementary material, which is available to authorized users.

## Background

The Gram-negative bacterium *Xanthomonas campestris* pathovar *campestris* (*Xcc*) is the causal agent of black rot disease, one of the most destructive diseases of cruciferous crops worldwide [1]. This pathogen can infect almost all members of the crucifer family (*Brassicaceae*), including many important vegetables, the major oil crop rape, and the model plant *Arabidopsis thaliana*. Over the past several decades, *Xcc* has been used as a model bacterium for studying molecular mechanisms of bacterial pathogenicity [2]. The entire genome sequences of a number of strains such as ATCC33913, 8004, and B100 have been determined [3–5] and a large number of genes associated with essential virulence have been identified. Among them, *rpf* (*r*egulation of *p*athogenicity *f*actors) and *hrp* (*h*ypersensitive *r*esponse and *p*athogenicity) clusters of genes are essential for pathogenicity of *Xcc* [6–8].

The *Xcc rpf* cluster of genes consists of at least nine genes (*rpfA* to *rpfI*). This gene cluster is involved in the quorum sensing system, controlling the synthesis of a diffusible signal factor (DSF) and regulating extracellular plant cell wall-degrading enzymes and extracellular polysaccharide (EPS) production as well as biofilm formation [6, 9–11]. The role of *rpfC*, *rpfF* and *rpfG* genes has been extensively studied [9–17]. The *rpfF* gene encodes an enzyme responsible for synthesizing the DSF molecules, which are secreted into extracellular environment [16]. The proteins encoded by *rpfC* and *rpfG* compose a two-component signal transduction system which is implicated in DSF perception and signal transduction [9, 12, 13]. RpfC acts as the histidine kinase sensor in the two component regulatory system to sense the environmental DSF signal, leading to activation of RpfG as a cyclic di-GMP phosphodiesterase. The activation of RpfG then leads to a reduction of cyclic di-GMP level which promotes synthesis of extracellular enzymes and EPS [9, 12, 13]. In addition, it is known that cyclic di-GMP effects on the synthesis of extracellular enzymes and EPS involve the transcriptional activator Clp (cAMP receptor-like protein). Cyclic di-GMP binds to Clp, thus preventing binding of Clp to the promoters of target genes that include those encoding extracellular enzymes and EPS biosynthesis [13–17].

In addition to the *rpf*/DSF regulatory system, the pathogenicity of *Xcc* is also dependent on the *hrp* cluster of genes. The *hrp* genes are associated with pathogen-induced hypersensitive response (HR), a disease-resistant phenomenon at the infection sites of resistant hosts and nonhost plants, and pathogen’s pathogenicity in susceptible hosts. Most *hrp* genes in the cluster encode the type III secretion system (T3SS) that translocates effector proteins into host cells and is highly conserved among Gram-negative pathogenic bacteria [18–20]. In *Xcc*, the *hrp* cluster is composed of six main operons (*hrpA* to *hrpF*) which harbor more than 20 different genes [7]. The expression of the operons is regulated by the AraC-type transcriptional activator HrpX [21]. The expression of *hrpX* is positively regulated by a two-component signal transduction system composed of HpaS and HrpG [21, 22]. HpaS is a histidine kinase sensor and HrpG is an OmpR family response regulator [22]. It is clear that the expression of the *hrp* genes including the regulators *hrpG* and *hrpX* is expressed at low levels in nutrient rich media but induced in plant tissues or in certain minimal media [7, 21].

As the *hrp* genes are induced in minimal media but expressed at low levels in nutrient rich media, the studies on the *hrp*/T3SS system were commonly carried out in certain minimal media. On the contrary, the *rpf*/DSF system is studied in nutrient rich media. To our knowledge, no work on the link between *rpf*/DSF and *hrp*/T3SS systems has been reported. The aim of this work was to identify upstream regulators of *hrpX* in *Xcc*. We employed the *sacB* strategy [23] to screen mutations that affect the expression of *hrpX*. Interestingly, we found that a mutation in the *rpfC* gene of the *rpf*/DSF system significantly reduced the expression of *hrpX*. Here, we provide evidences showing that RpfC positively regulates *hrpX*.

## Methods

### Bacterial strains, plasmids and growth conditions

The bacterial strains and plasmids used in this work are listed in Table 1. *Xcc* strains were grown at 28 °C in nutrient rich medium NYG [24] or minimal media MMX (23.8 mM glucose, 3.87 mM sodium citrate, 15.1 mM (NH_4_)_2_SO_4_, 0.81 mM MgSO_4_, 23 mM K_2_HPO_4_, 44 mM KH_2_PO_4_, pH 7.0) [24] and XCM1 (20 mM succinic acid, 0.15 g/l casamino acids, 7.57 mM (NH_4_)_2_SO_4_, 1 mM MgSO_4_, 60.34 mM K_2_HPO_4_, 33.07 mM KH_2_PO_4_, pH 6.6) [25]. Antibiotics were used at the following final concentrations as required: ampicillin (Amp), 100 μg/ml; gentamycin (Gm), 10 μg/ml; kanamycin (Kan), 25 μg/ml; rifampicin (Rif), 50 μg/ml; and tetracycline (Tc), 15 μg/ml for *Escherichia coli* and 5 μg/ml for *Xcc*. *E. coli* strains were grown in Luria-Bertani medium (LB, per liter: tryptone 10 g, yeast extract 5 g, NaCl 10 g) at 37 °C. The triparental conjugation between *Xcc* and *E. coli* strains was performed as described by Daniels and associates [24]. Restriction enzymes and DNA ligase were used in accordance with the manufacturer’s instructions (Promega, Madison, Wisconsin, USA).

**Table 1:** Bacterial strains and plasmids used in this work

Strains or plasmids	Relevant characteristics^a^	Source
*X. c.* pv. *campestris*		
8004	Wild type; Rif^r^	[24]
XB001	8004/pL6*hrpXsacB* with a Tn5 insertion in *XC_4007*; Rif^r^; Kan^r^; Tc^r^	This work
XB002	8004/pL6*hrpXsacB* with a Tn5 insertion in the intergenetic region	This work
	between the ORFs *XC_1510* and *XC_1511*; Rif^r^; Kan^r^; Tc^r^	
XB003	8004/pL6*hrpXsacB* with a Tn5 insertion in *XC_2333*; Rif^r^; Kan^r^; Tc^r^	This work
XB004	8004/pL6*hrpXsacB* with a Tn5 insertion in *XC_1192*; Rif^r^; Kan^r^; Tc^r^	This work
XB005	8004/pL6*hrpXsacB* with a Tn5 insertion in *XC_3951*; Rif^r^; Kan^r^; Tc^r^	This work
XB006	8004/pL6*hrpXsacB* with a Tn5 insertion in *XC_0124*; Rif^r^; Kan^r^; Tc^r^	This work
8004/pL6*hrpXsacB*	8004 harboring plasmid pL6*hrpXsacB*; Rif^r^; Tc^r^	This work
*ΔrpfC*	*rpfC* in frame deletion mutant of 8004; Rif^r^	This work
*CΔrpfC*	*ΔrpfC* harboring plasmid pLC*rpfC*; Rif^r^; Tc^r^	This work
*ΔrpfG*	*rpfG* in frame deletion mutant of 8004; Rif^r^	[17]
*ΔavrBs1*	*avrBs1* in frame deletion mutant of 8004; Rif^r^; Gm^r^	[44]
8004/pGUS*hrpG*	8004 harboring plasmid pGUS*hrpG*; Rif^r^; Tc^r^	This work
*ΔrpfC*/pGUS*hrpG*	*ΔrpfC* harboring plasmid pGUS*hrpG*; Rif^r^; Tc^r^	This work
8004/pGUS*hrpX*	8004 harboring plasmid pGUS*hrpX*; Rif^r^; Tc^r^	This work
*ΔrpfC*/pGUS*hrpX*	*ΔrpfC* harboring plasmid pGUS*hrpX*; Rif^r^; Tc^r^	This work
*E. coli*		
JM109	*RecA1*, *endA1*, *gyrA96*, *thi*, *supE44*, *relA1 Δ*(*lac*-*proAB*)/F′ [*traD36*, lacI^q^, *lacZ Δ*M15]	[29]
Plasmids		
pUC19	Cloning vector; Amp^r^	[28]
pLAFR6	Broad host range IncP cloning cosmid; Tc^r^	[28]
pK18mobsacB	Suicide plasmid in *Xcc*; Mob^+^ Tra^−^; Kan^r^	[27]
pLGUS	pLAFR6 containing a 1832-bp *gusA* ORF (excluding ATG), Tc^r^	[31]
pL6*sacB*	pLAFR6 containing a 1419-bp *sacB* gene, Tc^r^	This work
pK*rpfCsacB*	pK18mobsacB containing the two flanking fragments of *rpfC*; Kan^r^	This work
pUCP*hrpG*	pUC19 containing *hrpG* promoter; Amp^r^	This work
pUCP*hrpX*	pUC19 containing *hrpX* promoter; Amp^r^	This work
pGUS*hrpG*	pLAFR6 containing *hrpG* promoter in frame fused with *gus* gene; Tc^r^	This work
pGUS*hrpX*	pLAFR6 containing *hrpX* promoter in frame fused with *gus* gene; Tc^r^	This work
pL6*hrpXsacB*	pLAFR6 containing *hrpX* promoter in frame fused with *sacB* gene; Tc^r^	This work
pLC*rpfC*	pLAFR6 containing the sequenced whole ORF of *rpfC*; Tc^r^	This work

^a^Amp^r^, ampicillin-resistant; Gm^r^, gentamicin-resistant; Kan^r^, kanamycin-resistant; Rif^r^, rifampicin-resistant; Tc^r^, tetracycline-resistant

### Screen for mutations affecting the expression of *hrpX*

In order to screen the genes influencing the expression of *hrpX*, the *sacB* system [26] was employed. The 1419-bp *sacB* gene without the start codon ATG was amplified from the plasmid pK18mobsacB [27] (Table 1) using the primer pair sacB-F/sacB-R (Table 2). After confirmation by sequencing, the amplified *sacB* gene was ligated into the plasmid pLAFR6 [28] (Table 1), yielding the recombinant plasmid pL6*sacB* (Table 1). The promoter of *hrpX* was then in-frame cloned into pL6*sacB*, generating the plasmid pL6*hrpXsacB*, in which the *sacB* gene is driven by the *hrpX* promoter (Table 1). The plasmid pL6*hrpXsacB* was introduced into *Xcc* wild type strain 8004 from *E. coli* by triparental conjugation, yielding the strain 8004/pL6*hrpXsacB* (Table 1). The bacterial cells of strain 8004/pL6*hrpXsacB* were treated to be competent status and mutated by the EZ-Tn5™ transposon using a commercial EZ-Tn5™ transposon kit (Epicentre Biotechnology), followed by selecting mutant colonies on the plates of MMX minimal medium containing Rif, Kan, Tc and 5% sucrose.

**Table 2:** Primers used in this work

Primer name	Primer sequence	Product length (bp)
For construction		
sacB-F	CCCTCTAGA ATCAAAAAGTTTGCAAAACAAG	
sacB-R	CCCGTCGAC AAATAAAAGAAAATGCCAATAG	1419
RpfC-1-FOR	ATTGCGCTGATCCTGGTCTACAC	
RpfC-1-REV	CGGGATCC AGACTTCATAGACGCCTCAGACG	553
RpfC-2-FOR	CGGGATCC CGTAGCAACGAATAGACCGC	
RpfC-2-REV	ACAGCGACGTGTTCAATCTGGGCG	665
PhrpG-F	GGGGAGCTC GGTGTTCGGCACGCAGATGCGC	
PhrpG-R	GGGTCTAGA GTCCATCACTCGCGCGCCCACG	590
PhrpX-F	GGGGAATTC CTGACGCATAGGGCTGGTTGGGGC	
PhrpX-R	GGGGGTACC CTGGAGGTGCTGCAGACCCTGTGG	677
For sequencing		
KAN-2 FP-1	ACCTACAACAAAGCTCTCATCAACC	
KAN-2 RP-1	GCAATGTAACATCAGAGATTTTGAG	
For qReal-time PCR		
XC0052F	ACAGATTGGTCTCGCAGGTC	104
XC0052R	GGCAATGCTCTGATCGGTCT	
XC0241F	AGCCGCATCCACGAAACGGA	92
XC0241R	AACAGCGCGGTGCGTCGTAA	
XC1553F	TTTTCCGGATGGCTCGAACA	108
XC1553R	AGGATGCAGACTGACCAAGC	
XC2004F	TTGAGGCGGCCATATCACTC	119
XC2004R	CCACACTGCCGATACACCTT	
XC2081F	AGGAAGTGCGGATGAACCTG	141
XC2081R	CGCCGAAACCATTTCGAGAC	
XC2602F	TCGAGGATCCGCAAACTACG	110
XC2602R	GACCGGCATCGAGGAAAAGA	
XC2994F	CTCCTGCCATCTTGAGCGAT	122
XC2994R	CGCAATCAGCATGAAGTCCG	
XC2995F	CACGTGGGGCGAGAAAGATA	116
XC2995R	GCCGTTGGAACAAGGGAGTA	
XC3160F	GCTCGCAAGTCTGATGGAGT	126
XC3160R	CATGACGACAGACCCAGCTT	
XC3177F	ATGGACTCAGCGTTGTGGAG	110
XC3177R	TCATTGTTTCGTGGCAAGCG	
XC3802F	TTTCGACGATCTTCCCGAGC	111
XC3802R	TGGATGGAGGTGTTGTACGC	
XC4273F	CGGCGCGGAGTTAAATCTTG	129
XC4273R	AAAGTCTGCTCCGGGAATCG	
XC3076F	CGAAGTCGCATTGCTGGGCG	93
XC3076R	GCCTTGGACGCCTGCCGATA	
XC3077F	TGCGTGGCATCGGACGACAG	92
XC3077R	CACTCGAAACGGCCCAGCAC	
XC3002F	CCTGCAGACGATGGGCATCG	188
XC3002R	CGTCCTGTTGACCGCTCTGC	
XC3003F	CGTTACCTGATGACGCGCGT	155
XC3003R	AGGTCGGCGGATGCATAACC	
XC3004F	GCCTGGTGGGGCTGGTGTTCAA	164
XC3004R	CGTGCTCTGCTCACCGCTCA	
XC3005F	TGCAGCAGCTGAAGACGCGC	200
XC3005R	CAGGATCGCCTCGATGCCGA	
XC3006F	CGCCGTTTGGCGAGCTGGTGGG	179
XC3006R	CGCCTGCGCCTGGATCTGCA	
XC3007F	GCAGGCGCTGGCGGACGTCC	169
XC3007R	CACGCCGCGCTCGTTCCACG	
XC3008F	CCGTGTCCACGCTGGCGCAA	150
XC3008R	CGCCGACCTGCATGCTCGCC	
XC3009F	ACGGCCGGTGTGGATGCAGA	177
XC3009R	GGGTGTGGAGATCAGGCCGT	
XC3010F	GCTGATGCAATCCTCCTGCC	151
XC3010R	CCCCATCTTTGGCGCATTGG	
XC3011F	GCGAGTACTGCGGCCAGAGT	153
XC3011R	CAACACGCGTACAAGGCCTT	
XC3012F	TTGTGCAGACCGGGCTTAAT	160
XC3012R	TACCACAGCACCACGCCGAT	
XC3014F	GGATTGCCGGACACGGTGGT	150
XC3014R	TCGGGCGATCTGTCGACGAT	
XC3015F	TGGAACCACTGGGACTAGGCG	159
XC3015R	CAGCGCTAGCCGTTTGCAGC	
XC3016F	AATGCCATCGGCGTGCAGCA	172
XC3016R	CGCGACAGGCATCGAGCAAT	
XC3017F	GTGCGATTCACTTCCGAAGC	155
XC3017R	ACCACCACCAGCTTGAGCGC	
XC3018F	GAACTGGAAGAAGCCGAAGCG	192
XC3018R	ACGGGCGCTGTCGTCTACCT	
XC3019F	AGATTGGCCTGATTGTTCGC	178
XC3019R	CTCCAGCAGCGCAACATCGT	
XC3020F	CACGCTCACCCAGGATATGA	163
XC3020R	GACAATGAAATCGTTGCGCG	
XC3021F	GATTGGGCCAGGCCAGGGAT	168
XC3021R	CGTTCTTCTTCGCGGTCAGG	
XC3022F	CACATGCCTGCAGCCCAGAC	154
XC3022R	CCTGTGCGTACACCGACAAA	
XC3023F	CGCGCCACCCGGCCTCCAGA	185
XC3023R	CGCCGCCGCCCTTCATGTTG	
XC3024F	GTGCTGGGCCGTCACATGCT	155
XC3024R	ACCGCCTGCTGCACGACCGT	
XC3025F	GTTGCCGCCTGCGGTGGATG	188
XC3025R	GCAAGCCTTGCAGCGCACTC	
XC1331F	TGTGCCTGGATTCGGGTTGC	323
XC1331R	CCACCATCGGAAACTTGTCG	
XC3907F	CGATGTTCGCCACCCACAAC	318
XC3907R	GGATGGACGCAAACGAGGAC	
XC3379F	CAACGATGCGTCCAATGTGTC	301
XC3379R	CAAGGTTTCCACCGCTGCTG	
XC1969F	CGGCTACAAGAACGCCTACCCG	156
XC1969R	GCGATGTCCTGCTCGGAAAAGC	
XC2272F	GAGCCCTGAAATCGCCCTGACC	223
XC2272R	CTCCACCAGATGTCCCAGCAGC	
XC3324F	GTCTTCACTGCCGACGGTTC	164
XC3324R	TCGAATGCGACCTTCTCGATAC	
XC4122F	TTCGTATGATTTCCTCGGCC	142
XC4122R	TACTTGATCTTGCCTTCCTTGT	
XC1019F	ACACGATTTCTGGGTTTTGCGC	304
XC1019R	ATTCAGTGCGTTGAGTTCTGGC	
XC3862F	AGGCAAGCCCCGAATCCGAAGC	251
XC3862R	CACGGCGTCGTCCAGTGTGTTG	
XC4147F	ACGGCTACATCGGGTTGATC	197
XC4147R	TCATTTGCGGGCTTCCTCC	
XC2979F	ATGAGCGACTGGGAAGGACG	231
XC2979R	GGCAAACTGCTTGAGGTCAG	
XC0109F	GCGAAAAACGCCTGGCGGTGC	166
XC0109R	AGCTTGCCGGCATCCAGCGC	
XC0705F	CTACTGGCGTGACGTTGGTG	156
XC0705R	CACCCATCACACCGGACCTG	
XC1002F	CACTGCGTTATGTGCTGCCC	158
XC1002R	CAGTTTCGACGCGGCAATGG	
XC1850F	GGCAGCACGCGCCGCTACATCAG	151
XC1850R	TGGGCGTGGGGTTGGCATTG	
XC2254F	GAACTGGAACGTTGCCTGGG	150
XC2254R	GTGCGATGTCGCGACGAAGC	
XC1621F	GATCTGTGGAAGCAGTAACG	159
XC1621R	CTACTCGGGCCTTGAACAAC	
XC2512F	CGCGTGCGCGTAACGGTGTG	150
XC2512R	CGCTACGCGTGAAGCTGGGG	
XC0155F	GCGTGTTGCGCAGCTTCGAAC	168
XC0155R	GCATGCGCATCAGCTTGAGG	
XC1978F	CTCAAGCTGCGCGGCCATCC	151
XC1978R	GCACCATTGCGCGCCCCAGC	
XC1294F	GCGCGCAGCCAGTGCCGTGG	131
XC1294R	CGGTGCCGGCGACTGCCACT	
XC2088F	GCGAGTGGAAAAACCAGCTGGGT	140
XC2088R	AACCGGGTTGGCAAACCAGC	
XC3540F	TGAGCGTGCCAACAAGGACT	152
XC3540R	ATTCGACCTTGGTGCGCAGC	
XC3697F	GGCGACAGGCCCGCGGATGGTTGT	144
XC3697R	GCCCGCAGGCCCAGCCGAAT	
16S-F	GAGGAAGGTGGGGATGACGTCA	108
16S-R	GATTGGCTTACCCTCGCGGG	

To map the transposon insertion sites in the obtained mutants, the total DNA of each mutant was isolated and digested with *Eco*RI (no *Eco*RI site within the transposon), and then cloned into the plasmid pUC19 [29] (Table 1). The resulting recombinant plasmid was transformed into *E. coli* strain JM109 [29] (Table 1) and transformants were selected by Kan (for the transposon) plus Amp resistance. The recombinant plasmid was isolated from the obtained Kan- and Amp-resistant transformants and the DNA sequences flanking the transposon were identified by sequencing the recombinant plasmid using the primers KAN-2 FP-1 or KAN-2 RP-1 (Table 2).

### Construction of mutants and GUS reporters

An *rpfC* deletion mutant was generated by the methods described previously [30]. Briefly, two DNA fragments flanking *rpfC* gene were generated by PCR using the primer pairs RpfC-1-FOR/RpfC-1-REV and RpfC-2-FOR/RpfC-2-REV (Table 2). The resultant DNA fragments were cleaved with *Bam*HI and ligated. The fusion fragments were then amplified using the ligation mixture as the template and the primer pair RpfC-1-FOR/RpfC-2-REV and cloned into the *Sma*I site of vector pK18mobsacB and transformed into *E. coli* strain JM109. After sequence verification, the obtained recombinant plasmid was mobilized into *Xcc* strain 8004 by triparental conjugation. Transconjugants were firstly selected on NYG medium supplemented with Rif and Kan. The second selection was made on NYG medium containing 5% sucrose and Rif for resolution of the vector by a second crossover event. The in-frame deletion of *rpfC* was confirmed by PCR and sequencing.

To construct *Xcc hrpG* and *hrpX* promoter-*gusA* transcriptional fusion reporters, the promoter regions of *hrpG* and *hrpX* were amplified from *Xcc* strain 8004 using the primer sets PhrpG-F/PhrpG-R and PhrpX-F/PhrpX-R (Table 2), respectively. The amplified *hrpG* promoter fragment and *hrpX* promoter fragment were double digested with *Sac*I plus *Xba*I and *Eco*RI plus *Kpn*I, respectively, then ligated into the plasmid pUC19 (Table 1). The resulting recombinant plasmids were then transformed into *E. coli* JM109. Transformants were selected on LB medium supplemented with IPTG, X-gal (5-Bromo-4-chloro-3-indolyl-β-D-galactoside) and Kan. The positive colonies were confirmed by PCR and sequencing, generating the plasmids pUCP*hrpG* and pUCP*hrpX* (Table 1). The promoter regions of *hrpG* and *hrpX* were excised from plasmids pUCP*hrpG* and pUCP*hrpX* and cloned into pLGUS [31] (Table 1) and transformed into *E. coli* JM109. Transformants were selected on LB medium supplemented with Tc. Recombinant plasmids were isolated from the obtained transformants and confirmed by PCR and restriction enzyme digestion. The confirmed recombinant plasmids were named pGUS*hrpG* and pGUS*hrpX*, respectively. These reporter plasmids were subsequently transferred into *Xcc* strains *ΔrpfC* and 8004 by triparental conjugation. Transconjugants were selected on NYG medium supplemented with Rif and Tc. The resulting transconjugants 8004/pGUS*hrpG*, *ΔrpfC*/pGUS*hrpG*, 8004/pGUS*hrpX*, and *ΔrpfC*/pGUS*hrpX* (Table 1) were further confirmed by PCR and restriction enzyme digestion.

### HR test and electrolyte leakage assay

HR test was performed as described previously [32]. The *Xcc* nonhost plant pepper ECW-10R (*Capsicum annuum* cv. ECW-10R) was used. Pepper seedlings were grown in a greenhouse with 12 h day and night cycle illumination by fluorescent lamps at temperatures of 25 to 28 °C. Bacterial cells of *Xcc* strains from overnight cultures were washed and diluted to a concentration at an optical density at 0.01 (600 nm) (1 × 10^7^ CFU/ml) in 10 mM sodium phosphate buffer (5.8 mM Na_2_HPO_4_ and 4.2 mM NaH_2_PO_4_, pH 7.0) and approximately 5 μl bacterial suspension was infiltrated into the pepper leaf tissues at the stage of four fully expanded leaves using a needleless syringe. After infiltration, the plants were grown at 28 °C with a 16 h photoperiod per day and 80% relative humidity. HR symptoms were photographed at 8, 16, and 24 h post-inoculation. At least three plants were inoculated in each experiment, and each experiment was repeated at least three times.

For electrolyte leakage assay, bacterial suspensions were diluted to a concentration of OD_600_ = 0.01 in 10 mM sodium phosphate buffer and measurements were carried out exactly as described previously [33]. Essentially, for each sample, four leaf disks were removed with a 0.7-cm diameter cork borer, submerged in 10 ml of distilled water, and vacuum-infiltrated. Then, the net leakage after 1 h was measured with a conductivity meter (DDS-307A). Three samples were taken for each measurement in each experiment; the experiments were repeated at least twice.

### GUS activity assay

*Xcc* cells from overnight culture in NYG medium were resuspended in XCM1 medium to a final optical density of 0.1 (600 nm) and incubated for 24 h. Then, 1 ml of the culture was transferred to another 10 ml fresh XCM1 medium and incubated for 24 h. To determine the *β*-glucuronidase (GUS) activity of the bacterial cells, 200 μl cultures for each strain were mixed with 40 μl methylbenzene and vortexed. The supernatant was then taken for GUS activity assay. The GUS activity assay was performed by measurement of the OD_415_ using *ρ*-nitrophenyl*-β*-D-glucuronide as substrate as described previously [34].

### Histochemical GUS staining

Chinese radish cv. Manshenhong seedlings with four fully expanded leaves were used for inoculation. Histochemical GUS staining was performed by using 5-bromo-4-chloro-3-indolylglucuronide (Promega) as a substrate as described previously [34]. Bacterial suspensions of *Xcc* strains were diluted to a concentration of OD_600_ = 0.01 in sterile water and introduced into host plant leaves. For GUS activity quantification of bacterial cells in the plant leaves, the fluorogenic substrate 4-methylumbelliferyl-*β-*D-glucuronide was used following the method described previously [35]. For plant protein extraction, 10 mg plant leaves were added to 1 ml of cold GUS extraction buffer [50 mM Na_3_PO_4_, pH 7.0, 10 mM *β*-mercaptoethanol, 10 mM EDTA, 0.1% (*w*/*v*) sodium lauryl sarcosine, and 0.1% (*w*/*v*) Triton X-100] and grinded with mortar and pestle until homogenized. Then, 30 μl 0.1% SDS and 60 μl chloroform were added. After 10 s vortexes, samples were transformed into micro-centrifuge tubes and centrifugalized for 8 min at 8000 rcf. The plant extract protein was quantified and immediately tested by adding the GUS assay buffer [2 mM 4-MUG (4-Methyl-umbelliferyl-*β*-D-Glucuronide)]. The assay was performed using 5-bromo-4-chloro-3-indolylglucuronide (X-Gluc) (Promega) as substrate, essentially as described previously [35]. At least four wells for each concentration of MUG (two with plant extract and two with extraction buffer to serve as blanks and correct for any nonenzymatic hydrolysis of MUG). Final MUG concentrations of 10 μM, 30 μM, 50 μM, 70 μM, and 90 μM were used for plotting a standard curve. A 30 μM MUG was chosen to react with samples and the final volume was 100 μl. The plate was incubated at 37 °C for 10 min and then removed from heat and sat at room temperature for 2.5 h. Then, 200 μl of 0.2 M carbonate stop buffer was added to each well. Fluorescence was determined with emission and excitation filters set at 465 nm and 360 nm, respectively. The values for each time interval were averaged after subtracting the blank.

### Transcriptome analysis

*Xcc* cells from overnight culture in NYG medium were collected, washed twice with MMX medium and then transferred to 10 ml fresh MMX medium to a final optical density of 0.3 (600 nm) and incubated till the concentration up to OD_600_ = 0.6. The total RNA was extracted from the cultures with SV Total RNA Isolation System (Promega). RNA samples were quantified and qualified by Agilent Bioanalyzer (Agilent Technologies). The RNA integrity number (RIN) of total RNA should be greater than 8.0 and the rRNA ratio (23S/16S) should be greater than 1.2. The total RNA samples were digested by RQ DNase I (Promega) with a concentration of 1 U/μg of RNA samples. The RNA samples for transcriptome analysis were prepared according to the manufacturer’s manuals (Illumina). Briefly, rRNA was cleaned by Ribo-Zero™ rRNA Removal Kit (Gram-Negative Bacteria) (Epicentre Biotechnologies). After purification, the mRNA was fragmented into small pieces for first strand cDNA synthesis using the fragment agent (divalent cations) under elevated temperature. The synthesized cDNA fragments were added with adapters at their ends by an end repair process. The obtained products were purified and enriched with PCR to create the final cDNA libraries. The quality of these cDNA libraries was assessed using the Agilent Bioanalyzer and ABI Step One Plus Real-Time PCR (Applied Biosystems). The RNAs were sequenced by the Illumina sequencing platform (HiSeq 2000) in Beijing Genomics Institute at Shenzhen (BGI).

### Analysis of sequence data

The raw reads generated from the sequencing were cleaned up and mapped to the reference genomic sequence of *Xcc* strain 8004 by SOAP2/SOAP aligner [36]. The expression levels were evaluated by reads per kilobase per million mapped reads (RPKM) [37], which normalizes the reads count to the gene expression level by taking account of the gene length and sequencing depth. The differential expression genes (DEGs) analysis was performed as described by Audic and Clavier [38], in which false discovery rate (FDR) was used to determine the threshold of *p*-value in multiple tests. In this study FDR < 0.001 was used as the threshold to judge the significance of gene expression difference. RNA sequencing data from four samples [*ΔrfpC*-1, *ΔrfpC*-2, *Xcc* 8004–1 (WT-1), *Xcc* 8004–2 (WT-2)] were grouped into four pairs (*ΔrfpC*-1/WT-1, *ΔrfpC*-1/WT-2, *ΔrfpC*-2/WT-1, and *ΔrfpC*-2/WT-2). The log2 fold change of RPKM of mutant vs. wild type was counted. The average of the log2 fold values of the four pairs was used to assess the differential expression genes with a stringent cutoff value of |log2-fold value| ≥ 1.0 and *p* value < 0.01. The RNA sequencing strategy for *ΔrpfG* was the same as *ΔrpfC*.

### qRT-PCR analysis

*Xcc* cells from overnight culture in NYG medium were collected, washed twice with MMX medium and transferred to 10 ml fresh MMX medium to a final optical density of 0.3 (600 nm) and incubated till the concentration up to OD_600_ = 0.6. The total RNA was extracted from the cultures with SV Total RNA Isolation System (Promega). The PrimeScriptTM RT reagent Kit with gDNA Eraser (Perfect Real Time) (TakaRa) was employed to fulfill the digestion of genomic DNA and the synthesis of cDNA. The obtained cDNA template was diluted to a final concentration of 5 ng/μl and 2 μl aliquot was used for qRT-PCR analysis. 16S rDNA gene was used for normalization in the qRT-PCR analysis. The primer sets for randomly selected ORFs, *hrp* genes, and type III effector genes were listed in Table 2.

## Results

### Identification of positive regulator candidates affecting *hrpX* expression by *sacB* strategy

**Fig. 1:**
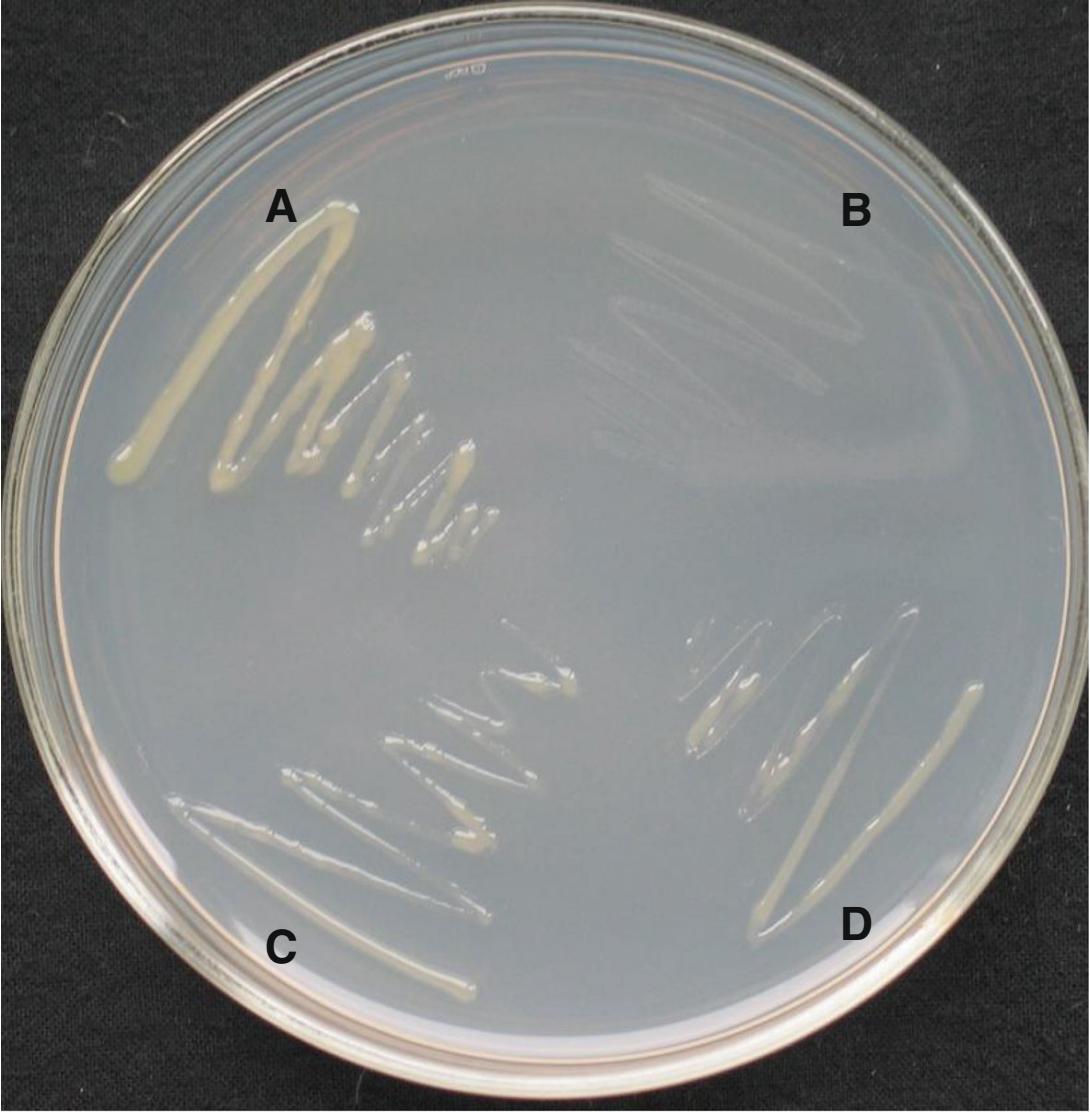
Identification of positive regulator candidates affecting *hrpX* expression by *sacB* strategy. *Xcc* wild type train 8004 and the deletion mutant strain *ΔhrpG* were used as controls. The principle in this strategy is that strain 8004/pL6*hrpXsacB* cannot grow on the minimal medium containing 5% sucrose, because the expression of the *hrpX*-promoter-driven *sacB* gene is lethal to the cells under these conditions, and only the strains with a mutation (i.e., deletion mutant of *hrpG*, *ΔhrpG*) impeding the expression of *hrpX* (i.e. strain *ΔhrpG*/pL6*hrpXsacB*, or disrupting the *sacB* gene, or the wild-type strain 8004 and the deletion mutant strain *ΔhrpG* can grow. **a**, wild-type strain 8004; **b**, 8004/pL6*hrpXsacB*; **c**, *ΔhrpG*/pL6*hrpXsacB*; **d**, the deletion mutant strain *ΔhrpG*

The *sacB* gene that encodes a levansucrase in *Bacillus subtilis* has been used as a tool for positive selection [23, 39–41]. The enzyme levansucrase catalyzes transfructorylation from sucrose to various acceptors, resulting in sucrose hydrolysis and the synthesis of levan, which is toxic to cells. It has been reported that expression of *sacB* gene in the presence of 5% sucrose in agar medium is lethal to a variety of bacteria including *E. coli*, *Agrobacterium tumefaciens*, and *Rhizobium meliloti* [23]. In this study, we found that similar to these bacteria, *Xcc* strain 8004 expressing *sacB* gene could not survive at the same sucrose concentration. Therefore, we used the *sacB* gene to screen candidates which positively regulate the expression of *hrpX*. In brief, firstly we constructed a recombinant plasmid pL6*hrpXsacB* (Table 1) by cloning a *sacB* gene into the broad host range plasmid pLAFR6 (Table 1), in which the *sacB* gene was driven by the promoter of *hrpX*. Then, the plasmid pL6*hrpXsacB* was transferred from *E. coli* into *Xcc* wild type strain 8004 by triparental conjugation. The obtained transconjugant strain 8004/pL6*hrpXsacB* (Table 1) was mutated by the EZ-Tn5™ transposon, followed by selecting mutant colonies on the plates of MMX minimal medium containing 5% sucrose. The principle in this strategy is that strain 8004/pL6*hrpXsacB* cannot grow on the minimal medium MMX containing 5% sucrose (Fig. 1b), because the expression of the *hrpX-*promoter-driven *sacB* gene is lethal to the cells under these conditions. However, the strains with a mutation (i.e., deletion mutant of *hrpG*, *ΔhrpG*) impeding the expression of *hrpX* (i.e. strain *ΔhrpG*/pL6*hrpXsacB*) (Fig. 1c) or disrupting the *sacB* gene and the wild-type strain 8004 as well as the deletion mutant strain *ΔhrpG* can grow (Fig. 1a and d).

Six mutants (named XB001 to XB006) (Table 1) were obtained in this work. The transposon insertion sites in these mutants were further mapped (see Methods for details), revealing that the mutations lie in the ORFs *XC_4007* (XB001), *XC_2333* (XB003), *XC_1192* (XB004), *XC_3951* (XB005) and *XC_0124* (XB006), and the intergenetic region between the ORFs *XC_1510* and *XC_1511* (XB002), respectively. Interestingly, the ORF *XC_2333* is the *rpfC* gene. The others were annotated to encode hypothetical proteins (*XC_4007* and *XC_1511*), antifreeze glycopeptide AFGP related protein (*XC_1192*), glucosyltransferase (*XC_3951*), TonB-dependent receptor (*XC_0124*), and TldD protein (*XC_1510*), respectively.

### RpfC positively regulates the expression of *hrpX*

**Fig. 2:**
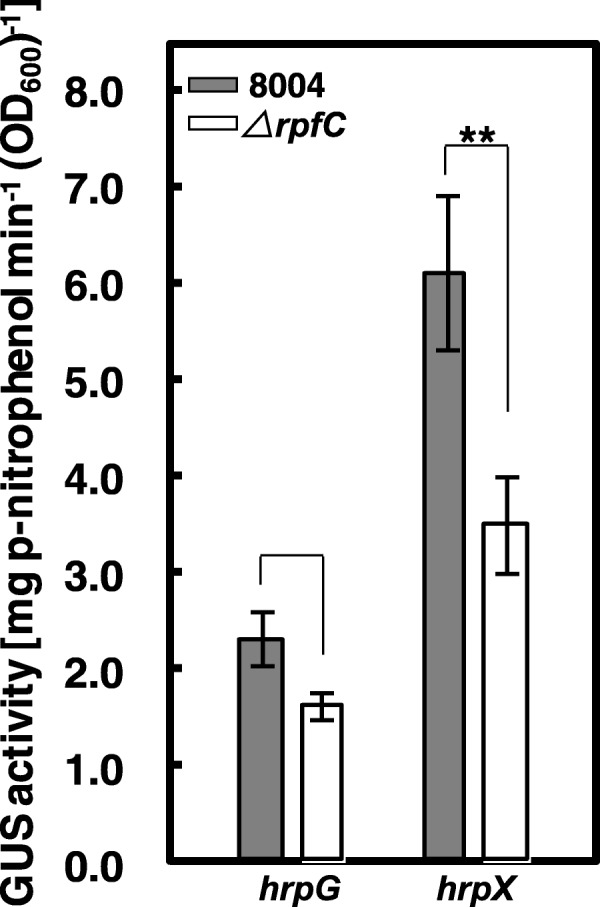
RpfC positively affects the expression of *hrpX* in XCM1 minimal medium. *β*-Glucuronidase (GUS) activities of *hrpG* and *hrpX* promoter-*gusA* reporters in the *rpfC* mutant and the wild-type backgrounds. Strains were cultured in XCM1 medium for 24 h, and GUS activities were then determined by measurement of optical density at 415 nm (OD_415_) using *ρ*-nitrophenyl*-β*-D-glucuronide as substrate. Data are mean ± standard deviations (SD) of triplicate measurements. The experiment was repeated twice and similar results were obtained. **, *t*-test, *p* < 0.01

As described above, RpfC is a key sensor kinase in *rpf*/DSF system. The above result suggests that RpfC may also play a role in the regulation of *hrp*/T3SS system. To further validate this result, we constructed a deletion mutant of *rpfC* (named *ΔrpfC*) and promoter-*gusA* transcriptional fusion reporter plasmids of *Xcc hrpG* and *hrpX* (named pGUS*hrpG* and pGUS*hrpX*) (see the Methods for details). The reporter plasmids were then transferred into the *rpfC* deletion mutant *ΔrpfC* and the wild-type strain 8004 by triparental conjugation, yielding reporter strains *ΔrpfC*/pGUS*hrpG*, *ΔrpfC*/pGUS*hrpX*, 8004/pGUS*hrpG*, and 8004/pGUS*hrpX*, respectively (Table 1). Subsequently, GUS activities of these strains grown in *hrp*-inducing minimal medium XCM1 were assayed. The results showed that the GUS activities of the strain *ΔrpfC*/pGUS*hrpX* was significantly lower than that of the strain 8004/pGUS*hrpX* (*p* = 0.005 by *t* test) (Fig. 2). Although the GUS activity of strain *ΔrpfC*/pGUS*hrpG* was lower than that of strain 8004/pGUS*hrpG*, their difference was not significant (*P* = 0.3344 by *t* test) (Fig. 2). These data suggest that RpfC is involved in positive regulation of the expression of *hrpX* and the regulation is probably independent of HrpG in the minimal medium XCM1.

**Fig. 3:**
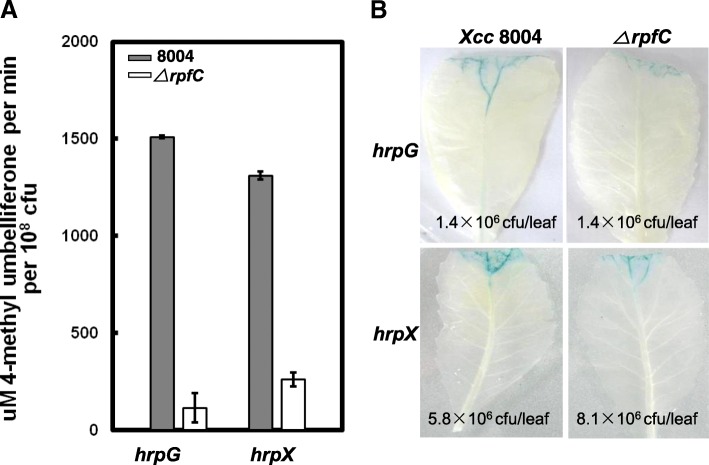
RpfC positively affects the expression of *hrpG* and *hrpX* in host plant. *Xcc* strains 8004/pGUS*hrpG*, 8004/pGUS*hrpX*, *ΔrpfC*/pGUS*hrpG*, and *ΔrpfC*/pGUS*hrpX* were cultured in NYG medium overnight and resuspended in water to an optical density at 600 nm of 0.01, and then inoculated into the Chinese radish cv. Manshenhong leaves by leaf clipping. At 5 days post-inoculation, the inoculated leaves were assayed. **a**, Leaves were taken and analyzed for bacterial numbers and GUS activity was measured with the fluorogenic substrate 4-methylumbelliferyl-*β*-D-glucuronide. GUS activity values per 10^8^ bacterial cells are the mean ± standard deviations of three independent measurements. **b**, GUS activity was measured using an in situ staining method, and bacterial cell numbers inside the infected leaves were measured in a parallel experiment. Average bacterial numbers inside the tested leaves are indicated. The experiments were repeated twice. Data presented are from a representative experiment, and similar results were obtained in the other independent experiment

To investigate whether RpfC regulates the expression of *hrpG* and *hrpX* in plants, the above reporter strains were inoculated into the host plant Chinese radish and the GUS activity in the inoculated levels were measured. As shown in Fig. 3, the strain *ΔrpfC*/pGUS*hrpX* produced significantly lower GUS activity compared to the strain 8004/pGUS*hrpX*, suggesting that RpfC positively regulates the expression of *hrpX* in planta. Interestingly, the strain *ΔrpfC*/pGUS*hrpG* also produced significantly lower GUS activity compared to the strain 8004/pGUS*hrpG* (Fig. 3). This indicates that RpfC regulates the expression of *hrpG* in planta. Taken together, these results imply that RpfC regulates the expression of *hrpX* in the minimal medium XCM1 as well as in the host plant Chinese radish and influences significantly the expression of *hrpG* in the host plant tissues but not in XCM1 medium.

### Mutation of *rpfC* results in a delayed and weakened HR induction

**Fig. 4:**
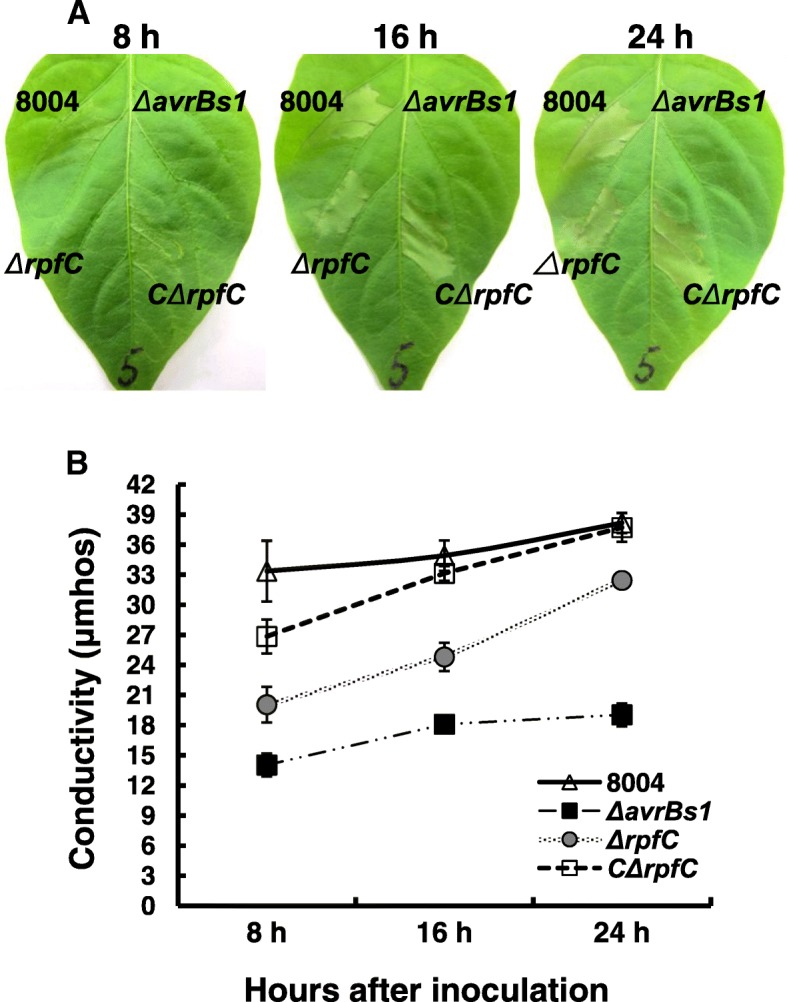
RpfC is involved in hypersensitive response. **a**, Hypersensitive response symptoms induced in pepper leaves (*Capsicum annuum* cv. ECW-10R) by the *Xcc* strains. Approximately 5 μl bacterial culture (1 × 10^7^ CFU/ml) suspended in 10 mM sodium phosphate buffer were infiltrated into the leaf mesophyll tissue with a blunt-end plastic syringe. Pictures of the pepper leaf were taken at 8, 16, and 24 h after infiltration. Three replications were done in each experiment, and each experiment was repeated three times. Results presented are from a representative experiment, and similar results were obtained in all other independent experiments. **b**, Electrolyte leakage from pepper leaves inoculated with *Xcc* strains. Results presented are from a representative experiment, and similar results were obtained in other independent experiments

The above results showed clearly that *rpfC* positively regulates the expression of the key regulator *hrpX* of the *hrp*/T3SS system. To verify whether mutation of *rpfC* affects the pathogen to induce HR on plants, the mutant strain *ΔrpfC* and the complemented strain C*ΔrpfC* (Table 1) were tested on *Xcc* nonhost pepper cultivar ECW-10R (*Capsicum annuum* cv. ECW-10R), which carries the resistance gene *Bs1* and has been typically used to test the HR of *Xcc* [33]. The experiment was carried out by infiltrating bacterial suspensions with a cell concentration of OD_600_ = 0.01 into the plant leaves. Strain *ΔavrBs1*, an *avrBs1-*deletion mutant of *Xcc*, which cannot elicit any HR symptoms on the pepper cultivar [42], was included as a negative control. Eight hours after inoculation, no significant HR phenotype was observed for the mutant strain *ΔrpfC*, while typical HR symptoms induced by the wild type strain 8004 and the complemented strain C*ΔrpfC* were observed (Fig. 4a). However, the mutant strain *ΔrpfC* produced visible HR symptoms 16 h after inoculation (Fig. 4a). These results were further substantiated using an electrolyte leakage assay. Both mutants (*ΔrpfC* and *ΔavrBs1*) showed significantly decreased electrolyte leakages at 8, 16, and 24 h after inoculation compared to the wild-type strain, although *ΔrpfC* showed stronger electrolyte leakage than *ΔavrBs1* (Fig. 4b). Consistent with the HR symptoms observed, the complemented strain and the wild type induced similar electrolyte leakages 16 h after inoculation (Fig. 4b). Taken together, these results reveal that RpfC is important for *Xcc* to stimulate a full HR on the nonhost plant pepper cultivar ECW-10R.

### RpfC and RpfG regulate the expression of a large set of genes in *Xcc* 8004

**Fig. 5:**
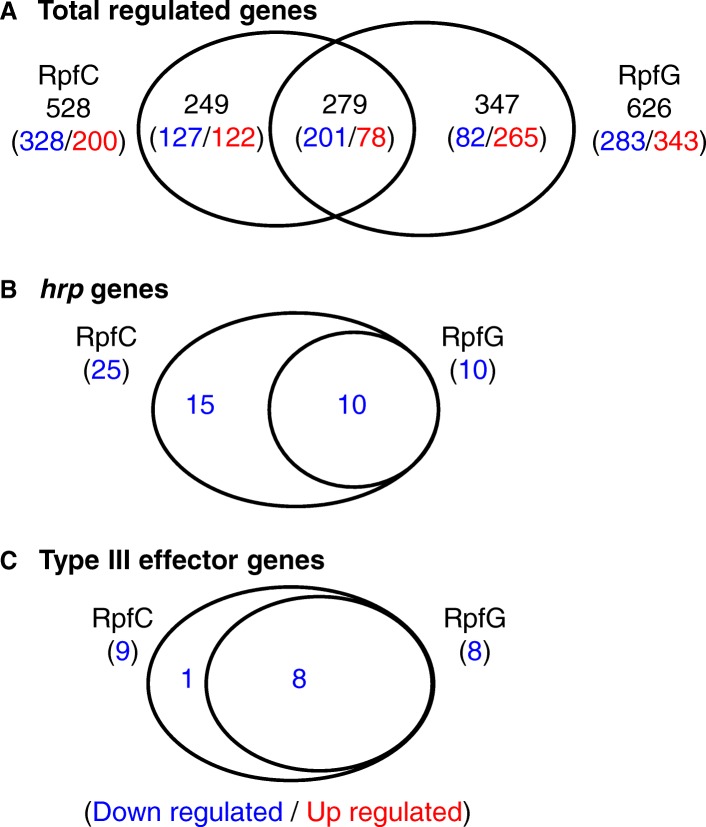
Comparison of RpfC and RpfG regulons. Venn diagrams showing the overlap of genes (**a**, Total regulated genes. **b**, *hrp *genes. **c**, Type III effector genes) whose expression is upregulated or downregulated in *rpfC* or *rpfG* deletion mutant backgrounds

**Fig. 6:**
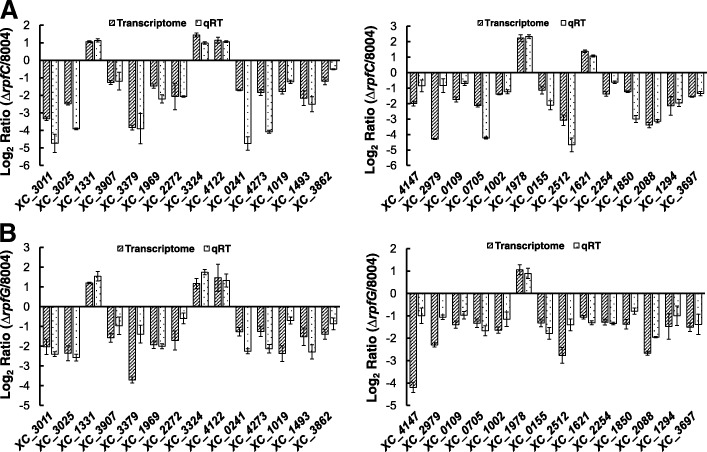
qRT-PCR verification of differently expressed genes in ΔrpfC (**a**) and ΔrpfG (**b**). The genes were chosen randomly from the transcriptome results. Two independent experiments were performed, and similar results were obtained. Results presented are from a representative experiment

To verify whether mutation of *rpfC* affects the expression of *hrp* genes via *rpfG* in minimal medium, the transcriptome of the mutant strains *ΔrpfC* and *ΔrpfG* were determined by RNA deep-sequencing. The mutant strains and the wild type strain 8004 were cultivated in the minimal medium MMX to a cell concentration of OD_600_ = 0.6–0.8. Total RNA was extracted from the cultures with SV Total RNA Isolation System (Promega). The RNA sequencing was carried out according to the manufacturer’s standard procedure (BGI). Through data analysis (Additional file 1: Table S1), a total of 528 RpfC-regulated genes were identified, among them 328 and 200 were down- and up-regulated, respectively; while 626 RpfG-regulated genes were identified, of which 283 and 343 were down- and up-regulated, respectively. Based on the published gene list of *Xcc* strain 8004 [4], the products of the RpfC- and RpfG-regulated genes could be grouped into the following 20 functional categories: (I) Nucleotide metabolism, (II) Carbohydrate metabolism, (III) Amino acid and protein metabolism, (IV) Chaperon and peptidases, (V) Fatty acid metabolism, (VI) Extracellular enzymes, (VII) Sugar kinase/transaminase, (VIII) Multidrug resistance and detoxification, (IX) Oxidative stress resistance, (X) Flagellum synthesis and motility, (XI) Hypersensitive reaction and pathogenicity, (XII) Iron uptake, (XIII) Ribosomal proteins, (XIV) Transcription regulators, (XV) Dehydrogenase, (XVI) Aerobic and anaerobic respiration, (XVII) Membrane components and transporters, (XVIII) Hypothetical proteins, (XIX) Environmental information processing, (XX) Others (Fig. 5, Additional file 2: Table S2 and Additional file 3: Table S3). To validate the transcriptome data, qRT-PCR was carried out. The result showed that the transcriptional expression of the 24 randomly selected genes, 2 *hrp* genes [*hrpB1* (*XC_3011*) and *hrpF* (*XC_3025*)], and 2 type III effector genes (*XC_0241* and *XC_4273*) was highly consistent with the transcriptome result (Fig. 6). A comparison of the genes regulated by RpfC and RpfG revealed that only 279 of them were regulated by both RpfC and RpfG (Fig. 5). This indicates that the regulons of RpfC and RpfG are not all the same.

### RpfC positively regulates 25 *hrp* genes, 9 reported T3S effector genes

The transcriptome result displayed that the expression of all the genes in the *hrp* cluster (*XC_3001*-*XC_3025*) and the regulator *hrpX* in *ΔrpfC* mutant cells was significantly (*p* ≤ 0.01 by *t-*test) lower than that in the wild type strain (Table 3). Furthermore, in *ΔrpfC* mutant cells the expression of the 9 reported T3S effector genes (*XC_0241*, *XC_1553*, *XC_2004*, *XC_2081*, *XC_2602*, *XC_2995*, *XC_3160*, *XC_3177*, and *XC_4273*) was also significantly (*P* ≤ 0.01 by *t-*test) lower than that in the wild type [3, 31, 42–44] (Table 3). However, the expression of *hrpG* and the global regulator *clp* in *rpf*/DSF system was not affected by the mutation of *rpfC* in the tested conditions (Table 3).

**Table 3:** RpfC positively regulates the expression of *hrpX*, 25 *hrp* genes, and 9 T3S effectors

ID	Gene name	Predicted product	Fold change	*p* value
XC3001	*hpa2*	Hpa2 protein	−1.967	0.006410439
XC3002	*hpa1*	Hpa1 protein	−3.429	5.28933E-05
XC3003	*hrcC*	HrcC protein	−2.440	6.32552E-05
XC3004	*hrcT*	HrpB8 protein	−2.112	0.001062566
XC3005	*hrpB7*	HrpB7 protein	−2.429	3.27619E-06
XC3006	*hrcN*	HrpB6 protein	−2.184	0.000117024
XC3007	*hrpB5*	HrpB5 protein	−3.356	1.38714E-05
XC3008	*hrpB4*	HrpB4 protein	−2.781	0.000112512
XC3009	*hrcJ*	HrcJ protein	−3.227	5.31033E-06
XC3010	*hrpB2*	HrpB2 protein	−3.152	5.78013E-05
XC3011	*hrpB1*	HrpB1 protein	−3.334	3.3299E-06
XC3012	*hrcU*	HrcU protein	−2.873	3.59286E-05
XC3013	*hrcV*	HrcV protein	−2.871	7.99441E-05
XC3014	*hpaP*	HpaP protein	−2.730	0.000117653
XC3015	*hrcQ*	HrcQ protein	−2.963	0.000143701
XC3016	*hrcR*	HrcR protein	−2.208	8.2237E-05
XC3017	*hrcS*	HrcS protein	−2.664	0.000432191
XC3018	*hpaA*	HpaA protein	−2.373	1.30182E-05
XC3019	*hrpD5*	HrpD5 protein	−2.843	2.26091E-05
XC3020	*hrpD6*	HrpD6 protein	−2.933	2.18335E-06
XC3021	*hrpE*	HrpE protein	−2.076	4.12178E-05
XC3022	*hpaB*	HpaB protein	−2.121	1.32695E-08
XC3023	*hrpW*	HrpW protein	−1.342	3.28466E-06
XC3024		conserved hypothetical protein	− 1.376	2.43557E-06
XC3025	*hrpF*	HrpF protein	−2.472	3.91605E-06
XC3076	*hrpX*	HrpX protein	−1.331	1.1147E-06
XC3077	*hrpG*	HrpG protein	−0.564	2.03168E-05
XC0052	*avrBs2*	avirulence protein	−0.556	0.000371266
XC0241	*xopXccN*	conserved hypothetical protein	−1.713	2.02564E-05
XC1553	*avrAC*_*Xcc8004*_	leucin rich protein	−1.796	6.64485E-05
XC2004	*avrXccC*	avirulence protein	−1.424	0.000257062
XC2081	*avrBs1*	avirulence protein	−1.357	0.00061082
XC2602	*avrXccE1*	avirulence protein	−1.458	1.49178E-06
XC2994	*xopXccP*	Type III effector protein	−0.626	0.000168654
XC2995	*xopXccE1*	Type III effector protein	−1.932	2.51053E-06
XC3160	*xopXccR1*	Type III effector protein	−2.954	1.98578E-05
XC3177	*xopXccQ*	Type III effector protein	−2.266	3.59482E-05
XC3802	*avrXccB*	avirulence protein	−0.449	0.000671213
XC4273	*xopXccLR*	leucin rich protein	−1.842	3.38357E-07
XC0486	*clp*	CAP-like protein	0.091	0.000208009

Fold change means the value of log2 ratio of RPKM (*ΔrfpC*/wild type). The differential expression genes were defined with a stringent cutoff value of |log2-fold change| ≥ 1.0 and *p* value < 0.01

Notably, the transcriptome analysis revealed that mutation of *rpfG* did not affect the expression of *hrpG, hrpX* and *clp* (Table 4), but significantly (*P* ≤ 0.01 by *t* test) influence the expression of some *hrp* genes (*XC_3009* to *XC_3015*, *XC_3019*, *XC_3021*, and *XC_3025*) and most of the reported T3S effector genes (*XC_0241*, *XC_2004*, *XC_2081*, *XC_2602*, *XC_2995*, *XC_3160*, *XC_3177*, and *XC_4273*) (Table 4). Given that RpfC and RpfG compose a two-component regulatory system, it is worthy to further study how they regulate the *hrp* and T3S effector genes. Nevertheless, these results reveal that RpfC positively regulates the expression of *hrp* and T3S effector genes as well as *hrpX* but not *hrpG* and *clp* in the minimal medium MMX.

**Table 4:** RpfG positively regulates the expression of 10 *hrp* genes, 8 T3S effectors

ID	Gene name	Predicted product	Fold change	*p* value
XC3001	*hpa2*	Hpa2 protein	−0.460	0.014094188
XC3002	*hpa1*	Hpa1 protein	−0.794	1.9328E-05
XC3003	*hrcC*	HrcC protein	−0.748	0.000323325
XC3004	*hrcT*	HrpB8 protein	−0.819	0.007692677
XC3005	*hrpB7*	HrpB7 protein	−0.898	0.000925861
XC3006	*hrcN*	HrpB6 protein	−0.866	0.001395029
XC3007	*hrpB5*	HrpB5 protein	−0.422	0.002457912
XC3008	*hrpB4*	HrpB4 protein	−0.604	0.000177562
XC3009	*hrcJ*	HrcJ protein	−1.370	0.000105572
XC3010	*hrpB2*	HrpB2 protein	−1.189	0.000499769
XC3011	*hrpB1*	HrpB1 protein	−2.031	0.000552365
XC3012	*hrcU*	HrcU protein	−1.364	1.2705E-05
XC3013	*hrcV*	HrcV protein	−1.251	0.000455787
XC3014	*hpaP*	HpaP protein	−1.270	0.000271481
XC3015	*hrcQ*	HrcQ protein	−1.055	0.002525553
XC3016	*hrcR*	HrcR protein	−0.969	0.003879682
XC3017	*hrcS*	HrcS protein	−0.999	0.032254632
XC3018	*hpaA*	HpaA protein	−0.511	0.000910631
XC3019	*hrpD5*	HrpD5 protein	−1.198	0.000505121
XC3020	*hrpD6*	HrpD6 protein	−1.141	0.000534484
XC3021	*hrpE*	HrpE protein	−1.388	0.000719991
XC3022	*hpaB*	HpaB protein	−0.589	0.000803494
XC3023	*hrpW*	HrpW protein	−0.214	9.24647E-05
XC3024		conserved hypothetical protein	−0.621	0.000308403
XC3025	*hrpF*	HrpF protein	−2.360	0.000402749
XC3076	*hrpX*	HrpX protein	0.034	4.24498E-05
XC3077	*hrpG*	HrpG protein	−0.105	0.000180844
XC0052	*avrBs2*	avirulence protein	0.037	0.002116633
XC0241	*xopXccN*	conserved hypothetical protein	−1.272	0.000227566
XC1553	*avrAC*_*Xcc8004*_	leucin rich protein	−0.942	0.000122936
XC2004	*avrXccC*	avirulence protein	−1.352	0.00359996
XC2081	*avrBs1*	avirulence protein	−1.786	0.002769123
XC2602	*avrXccE1*	avirulence protein	−1.512	0.000120947
XC2994	*xopXccP*	Type III effector protein	−0.970	0.001806466
XC2995	*xopXccE1*	Type III effector protein	−1.246	0.000429812
XC3160	*xopXccR1*	Type III effector protein	−2.452	0.000264107
XC3177	*xopXccQ*	Type III effector protein	−2.164	0.001441317
XC3802	*avrXccB*	avirulence protein	−0.562	0.002544406
XC4273	*xopXccLR*	leucin rich protein	−1.251	0.000444297
XC0486	*clp*	CAP-like protein	0.199	0.000155663

Fold change means the value of log2 ratio of RPKM (*ΔrfpG*/wild type). The differential expression genes were defined with a stringent cutoff value of |log2-fold change| ≥ 1.0 and *p* value < 0.01

## Discussion

The above results demonstrate that the sensor RpfC of the *rpf/*DSF cell-cell signaling system positively regulates the expression of the key regulator *hrpX* of the *hrp*/T3SS system in *Xcc*. Disruption of the *rpfC* gene in *Xcc* strain 8004 caused a significant decrease in the transcription of the *hrp* genes in minimal medium and host plant (Fig. 2, Fig. 3, Table 3, Table 4), resulting in a delayed and weakened HR (Fig. 4). The cell-cell signaling system is generally considered to facilitate gene expression when the bacterial population has reached a sufficient cell density [45]. Almost all of the previous studies on the *rpf*/DSF system of *Xcc* and its regulation in the synthesis of the virulence factors such as extracellular enzymes and EPS were carried out by growing bacterial cells in nutrient rich conditions to allow the bacterium to reach a high cell density. On the contrary, as the expression of *hrp* genes is repressed in nutrient rich media and induced in certain minimal media and plants, almost all of the studies on the *hrp*/T3SS system were carried out in minimal media or plants. The connection between these two systems has been neglected. We were lucky that *rpfC* gene was identified in the mutagenesis screen for *hrpX*-upstream regulatory genes.

Recent evidence suggests that perception of the DSF signal by RpfC leads to activation of RpfG as a phosphodiesterase that degrades cyclic di-GMP. Cyclic di-GMP is a second messenger which can bind to Clp to prevent binding of Clp to the promoters of target genes. The Clp regulator contains an N-terminal cNMP binding domain and a C-terminal DNA-binding domain. The decrease in cyclic di-GMP level by the phosphodiesterase activity relieves this inhibition, thus allowing Clp to bind to target promoter DNA sequences and activate target gene expression [13, 14, 46–48]. In a previous transcriptome profiling analysis in *Xcc* strain XC1 cultivated in a nutrient rich medium, it was found that mutation of *clp* affects the transcription of 299 genes. Within these Clp-regulated genes, 260 were up-regulated and 39 down-regulated. The latter genes include 9 *hrp* genes (*hrpB5*, *hrpD5*, *hrcR*, *hrpW*, *hpaP*, *hrpB2*, *hrpB7*, *hrpB4*, and *hpa1*) but neither *hrpG* nor *hrpX* [15]. These implied that RpfC regulates the expression of the *hrp* genes might via RpfG and the global transcriptional regulator Clp in *Xcc*. However, An and associates found that mutation of *rpfC* or *rpfG* in *Xcc* strain 8004 grown in the nutrient rich medium NYG did not affect the expression of *hrp* genes [49]. Our RNA sequencing data demonstrated that in minimal medium, RpfC positively regulates the expression of nearly all the *hrp* genes (Table 3) and RpfG controls some of the *hrp* genes (Table 4). These results indicate that RpfC and RpfG have different effects on the expression of the *hrp* genes in *Xcc* strain 8004 when grown in nutrient-rich and nutrient-deficient conditions. Our data also displayed that in minimal medium RpfC regulates the expression of *hrpX* but not *hrpG* and RpfG does not regulate the expression of both *hrpG* and *hrpX* (Table 3, Table 4). These results suggest that RpfC activate the expression of *hrpX* in minimal medium via neither RpfG nor HrpG. However, mutation of *rpfC* significantly reduced the expression of not only *hrpX* but also *hrpG in planta* (Fig. 3). This implies that RpfC regulates the *hrp* genes via different manners in minimal medium and host plants.

As mentioned above, it is known that the core regulatory mechanism in *Xcc rpf*/DSF quorum sensing system is RpfC-RpfG-c-di-GMP-Clp cascade. However, our transcriptome result showed that the regulons of RpfC and RpfG in the minimal medium MMX are not all the same. Similarly, the regulons of RpfC and RpfG of *Xanthomonas citri* subsp. *citri* in nutrient rich medium are also different [50]. These findings suggest that RpfC may regulate a number of genes independent of RpfG. Our data presented in this work show that RpfC may employ an undefined pathway other than the RpfC-RpfG-c-di-GMP-Clp cascade to regulate the expression of the *hrp* key regulator HprX in the minimal medium MMX. To further dissect how RpfC affects the expression of *hrpX* will be commendable. Interestingly, RpfC controls the expression of *hrpG* in host plants (Fig. 3). This suggests that the regulation net between the *rpf*/DSF and *hrp/*T3SS systems are rather complex. To further uncover this issue will be valuable.

## Conclusions

In this work, we found that mutation of the gene encoding the sensor RpfC of the *rpf*/DSF system significantly reduced the expression of *hrpX*, the key regulator of the *hrp*/T3SS system. Here, we provide evidences to demonstrate that RpfC positively regulates the expression of *hrpX* independent of RpfG, the cognate response regulator of RpfC, showing a complex regulatory network linking the *rpf/*DSF and *hrp*/T3SS systems.

## Additional files

Additional file 1: **Table S1.** RNA sequencing detail raw data. (XLS 8055 kb)

Additional file 2: **Table S2.** Functional groups of RpfC- regulated genes. (DOCX 19 kb)

Additional file 3: **Table S3.** Functional groups of RpfG- regulated genes. (DOCX 20 kb)

## Abbreviations

4-MUG: 4-Methyl-umbelliferyl-*β*-D-Glucuronide
Amp: Ampicillin
BGI: Beijing Genomics Institute
CFU: Colony forming unit
Clp: cAMP receptor-like protein
DEGs: Differential expression genes
DSF: Diffusible signaling factor
FDR: False discovery rate
Gm: Gentamycin
GUS: *β*-glucuronidase
HR: Hypersensitive response
*hrp*: Hypersensitive response and pathogenicity
Kan: Kanamycin
Rif: Rifampicin
*rpf*: Regulation of pathogenicity factors
RPKM: Reads per kilobase per million mapped reads
SD: Standard deviations
T3SS: Type III secretion system
Tc: Tetracycline
*Xcc*: *Xanthomonas campestris* pv. *campestris*
X-Gal: 5-Bromo-4-chloro-3-indolyl-β-D-galactoside
X-Gluc: 5-bromo-4-chloro-3-indolylglucuronide
