# Supplementary material for: RpfC regulates the expression of the key regulator hrpX of the hrp/T3SS system in Xanthomonas campestris pv. campestris
Source: BMC Microbiol. 2018 Sep 3;18:103. doi: 10.1186/s12866-018-1233-5 (PMC6122198; doi:10.1186/s12866-018-1233-5)
Supplement: Supplementary file 2 — Table S2. Functional groups of RpfC- regulated genes. (DOCX 19 kb) [file 12866_2018_1233_MOESM2_ESM.docx]

**Table S2.** Functional groups of RpfC- regulated genes.

| **Gene family** | **Gene ID** | **Fold change** |
| --- | --- | --- |
|  |  | **WT/*ΔrfpC*** |
| (I) Nucleotide metabolism (15) | *XC0109, XC0633, XC0888, XC0960, XC1332, XC1800, XC1808, XC2186, XC2192, XC2299, XC3157, XC3158, XC3967, XC4180, XC4203* | -2.69-2.88 |
| (II) Carbohydrate metabolism (68) | *XC0104, XC0142, XC0143, XC0150, XC0247, XC0318, XC0425, XC0645, XC0705, XC0809, XC0992, XC0993, XC0994, XC1002, XC1003, XC1005, XC1047, XC1166, XC1217, XC1218, XC1219, XC1291, XC1292, XC1331, XC1452, XC1470, XC1568, XC1582, XC1594, XC1596, XC1597, XC1598, XC1599, XC1645, XC1743, XC1744, XC1849, XC1873, XC1973, XC1975, XC1976, XC2087, XC2172, XC2188, XC2326, XC2458, XC2727, XC2834, XC2980, XC2981, XC3050, XC3054, XC3139, XC3159, XC3172, XC3415, XC3426, XC3427, XC3627, XC3751, XC3762, XC3763, XC3767, XC3769, XC3886, XC3907, XC3952, XC4154* | -5.07-3.55 |
| (III) Amino acid and protein metabolism (23) | *XC0031, XC0032, XC0165, XC0450, XC0473, XC0474, XC0548, XC0886, XC0889, XC1090, XC1232, XC1344, XC1569, XC1699, XC1871, XC1876, XC2374, XC2375, XC2376, XC3135, XC3456, XC3895, XC4056* | -3.21-2.14 |
| (IV) Chaperon and peptidases (5) | *XC0083, XC1335, XC2977, XC3156, XC3280* | -3.49-1.30 |
| (V) Fatty acid metabolism (1) | *XC2191* | -3.06 |
| (VI) Extracellular enzymes (17) | |  |
| Cellulases | *XC0783, XC0784* | -3.03--2.57 |
| Pectate lyases | *XC1298, XC1850* | -1.16--1.70 |
| Proteases | *XC1422, XC1447, XC1449, XC1450, XC1514, XC1515, XC3378, XC3379, XC3550, XC3575* | -5.03-3.10 |
| Lipases and Amylases | *XC0141, XC3487* | -3.55-1.26 |
| LPS and EPS | *XC2173* | -1.10 |
| (VII) Sugar kinase/transaminase (1) | *XC2254* | -1.38 |
| (VIII) Mutidrug resistance and detoxification (3) | *XC2306, XC3153, XC3724* | -2.33--1.00 |
| (IX) Oxidative stress resistance (10) | *XC0887, XC1454, XC1969, XC3081, XC3083, XC3084, XC3901, XC3902, XC3904, XC4152* | -4.53-1.69 |
| (X) Flagellum synthesis and motility (38) | *XC0325, XC0937, XC0938, XC0939, XC0940, XC0941, XC1184, XC1185, XC1186, XC1187, XC1358, XC1359, XC1621, XC1622, XC1624, XC1625, XC2160, XC2232, XC2234, XC2235, XC2236, XC2237, XC2239, XC2240, XC2246, XC2259, XC2260, XC2266, XC2267, XC2269, XC2270, XC2272, XC2277, XC2278, XC2279, XC2280, XC2298, XC2858* | -5.68-2.26 |
| (XI) Hypersensitive reaction and pathogenicity (37) | |  |
| Hrp related proteins | *XC3001, XC3002, XC3003, XC3004, XC3005, XC3006, XC3007, XC3008, XC3009, XC3010, XC3011, XC3012, XC3013, XC3014, XC3015, XC3016, XC3017, XC3018, XC3019, XC3020, XC3021, XC3022, XC3023, XC3024, XC3025, XC3076* | -3.53--1.43 |
| T3s effectors and virulence proteins | *XC0241, XC1553, XC2004, XC2081, XC2602, XC2995, XC3160, XC3177, XC4273 , XC2082, XC3182* | -3.12--1.45 |
| (XII) Iron uptake (16) | *XC1079, XC1115, XC1004, XC1165, XC1222, XC1241, XC1284, XC1451, XC1644, XC2137, XC2194, XC2296, XC2485, XC2512, XC3209, XC4053* | -4.04-1.75 |
| (XIII) Ribosomal proteins (26) | *XC1048, XC3092, XC3315, XC3319, XC3321, XC3322, XC3323, XC3324, XC3329, XC3330, XC3331, XC3333, XC3334, XC3335, XC3336, XC3337, XC3338, XC3339, XC3340, XC3341, XC3348, XC3349, XC3350, XC3351, XC3357, XC4122* | 1.44-2.26 |
| (XIV) Transcription regulators (15) | *XC0118, XC0689, XC0891, XC1435, XC1745, XC1909, XC2251, XC2281, XC2729, XC3316, XC3346, XC3347, XC3425, XC4055, XC4222* | -3.43-4.99 |
| (XV) Dehydrogenase (4) | *XC0443, XC0444, XC3774, XC3885* | -2.01--1.21 |
| (XVI) Aerobic and anaerobic respiration (12) | |  |
| Oxidases and oxidoreductase | *XC1385, XC1386, XC3138, XC3167, XC3170, XC3766* | -2.74-1.22 |
| Transferase | *XC0035, XC0209, XC0341, XC1648, XC3951, XC4294* | -2.95-1.58 |
| (XVII) Membrane components and transporters (46) | *XC0155, XC0196, XC0431, XC0470, XC0504, XC0810, XC0811, XC0819, XC0820, XC0893, XC0894, XC0969, XC0970, XC1087, XC1142, XC1345, XC1346, XC1459, XC1619, XC1626, XC1632, XC1633, XC1634, XC1635, XC1637, XC1639, XC1647, XC1682, XC1912, XC1913, XC1914, XC1978, XC2546, XC2728, XC2857, XC2859, XC3129, XC3294, XC3320, XC3576, XC3628, XC3771, XC4054, XC414, XC4224, XC4327* | -3.88-2.62 |
| (XVIII) Hypothetical proteins (171) | *XC0036, XC0105, XC0108, XC0117, XC0180, XC0230, XC0242, XC0262, XC0263, XC0268, XC0340, XC0347, XC0414, XC0426, XC0428, XC0442, XC0542, XC0543, XC0563, XC0624, XC0632, XC0634, XC0650, XC0710, XC0727, XC0776, XC0792, XC0815, XC0817, XC0880, XC0883, XC0980, XC1019, XC1020, XC1044, XC1045, XC1068, XC1106, XC1147, XC1188, XC1190, XC1210, XC1289, XC1294, XC1297, XC1353, XC1361, XC1401, XC1453, XC1458, XC1460, XC1493, XC1527, XC1581, XC1583, XC1623, XC1631, XC1646, XC1687, XC1732, XC1765, XC1807, XC1852, XC1853, XC1877, XC1910, XC1911, XC1915, XC1916, XC2009, XC2088, XC2135,*  *XC2164, XC2185, XC2187, XC2189, XC2190, XC2353, XC2410, XC2418, XC2419, XC2420, XC2424, XC2437, XC2474, XC2584, XC2587, XC2631, XC2632, XC2633, XC2634, XC2635, XC2726, XC2759, XC2773, XC2860, XC2861, XC2888, XC2898, XC2903, XC2921, XC2938, XC3000, XC3078, XC3127, XC3128, XC3147, XC3152, XC3171, XC3176, XC3207, XC3218, XC3253, XC3417, XC3540, XC3545, XC3549, XC3553, XC3554, XC3555, XC3556, XC3562, XC3695, XC3696, XC3697, XC3711, XC3715, XC3717, XC3721, XC3734, XC3744, XC3752, XC3753, XC3754, XC3755, XC3756, XC3761, XC3764, XC3765, XC3768, XC3770, XC3772, XC3773, XC3775, XC3820, XC3855, XC3856, XC3862, XC3863, XC3870, XC3882, XC3883, XC3899, XC3900, XC3903, XC3922, XC3955, XC3956, XC3961, XC3968, XC3969, XC3970, XC4012, XC4035, XC4147, XC4148, XC4153, XC4206, XC4245, XC4295, XC4312* | -8.67-4.86 |
| (XIX) Environmental information processing (11) | *XC0195, XC0197, XC0198, XC1290, XC1383, XC1755, XC1756, XC1938, XC1939, XC2163, XC3758* | -2.48-2.34 |
| (XX ) Other proteins (9) | *XC1640, XC2110, XC2438 , XC2636, XC2978, XC2979, XC4291, XC4292, XC4293* | -4.33-1.29 |
| Total gene number | *528* |  |

**Notes:** Ratio means the value of log2 ratio of RPKM (mutant / wild type).
