# Supplementary material for: RpfC regulates the expression of the key regulator hrpX of the hrp/T3SS system in Xanthomonas campestris pv. campestris
Source: BMC Microbiol. 2018 Sep 3;18:103. doi: 10.1186/s12866-018-1233-5 (PMC6122198; doi:10.1186/s12866-018-1233-5)
Supplement: Supplementary file 3 — Table S3. Functional groups of RpfG- regulated genes. (DOCX 20 kb) [file 12866_2018_1233_MOESM3_ESM.docx]

**Table S3.** Functional groups of RpfG- regulated genes.

| **Gene family** | **Gene ID** | **Fold change** |
| --- | --- | --- |
|  |  | **WT/*ΔrfpG*** |
| (I) Nucleotide metabolism (15) | *XC0001, XC0109, XC0509, XC0705, XC0908, XC1234, XC3079, XC3157, XC3208, XC3316, XC3354, XC3383, XC3643, XC4038, XC4180* | -1.52-2.73 |
| (II) Carbohydrate metabolism (75) | *XC0014, XC0020, XC0031, XC0032, XC0051, XC0104, XC0142, XC0143, XC0150, XC0158, XC0193, XC0374, XC0553, XC0645, XC0753, XC0754, XC0809, XC0851, XC0992, XC0993, XC1002, XC1003, XC1005, XC1047, XC1095, XC1214, XC1217, XC1218, XC1219, XC1233, XC1452, XC1470, XC1645, XC1849, XC2176, XC2188, XC2192, XC2324, XC2357, XC2458, XC2469, XC2477, XC2478, XC2482, XC2537, XC2637, XC2809, XC2871, XC2885, XC2977, XC2978, XC2979, XC2980, XC2981, XC2984, XC3045, XC3054, XC3158, XC3159, XC3172, XC3359, XC3426, XC3427, XC3433, XC3644, XC3769, XC3885, XC3895, XC3952, XC4096, XC4098, XC4149, XC4154, XC4158, XC4315* | -5.08-2.17 |
| (III) Amino acid and protein metabolism (15) | *XC0474, XC0481, XC0548, XC1344, XC1367, XC1871, XC1873, XC2375, XC2376, XC2466, XC2894, XC3456, XC3907, XC4056, XC4225* | -2.16-2.41 |
| (IV) Chaperon and peptidases (28) | *XC0227, XC0240, XC0253, XC0493, XC0610, XC0636, XC0643, XC0654, XC0997, XC1007, XC1008, XC1096, XC1331, XC1335, XC1544, XC1725, XC1881, XC2538, XC2826, XC3151, XC3156, XC3192, XC3201, XC3280, XC3502, XC3541, XC4073, XC4105* | -1.78-2.85 |
| (V) Fatty acid metabolism (1) | *XC2907* | -1.62 |
| (VI) Extracellular enzymes (30) | |  |
| Cellulases | *XC0027, XC0639, XC0783, XC0784* | -5.15-1.02 |
| Pectate lyases | *XC1298, XC1850, XC3591* | -2.24--1.57 |
| Proteases | *XC1291, XC1292, XC1422, XC1447, XC1449, XC1450, XC1514, XC1515, XC1859, XC3378, XC3379, XC3550, XC3575, XC3832* | -5.29-2.45 |
| Lipases and Amylases | *XC0276, XC1017, XC3487* | -3.93-2.22 |
| LPS and EPS | *XC1498, XC1658, XC1659, XC1667, XC1668, XC4013* | -2.14-1.61 |
| (VII) Sugar kinase/transaminase (2) | *XC1978, XC2254* | -1.41-1.33 |
| (VIII) Mutidrug resistance and detoxification (7) | *XC0274, XC1482, XC1754, XC1865, XC2306, XC3860, XC4256* | -2.33-2.67 |
| (IX) Oxidative stress resistance (13) | *XC0200, XC0201, XC0977, XC1454, XC1969, XC2564, XC3081, XC3083, XC3901, XC3902, XC3904, XC3905, XC4152* | -5.00-2.69 |
| (X) Flagellum synthesis and motility (25) | *XC0325, XC2115, XC2160, XC2234, XC2235, XC2236, XC2237, XC2239, XC2240, XC2246, XC2259, XC2260, XC2266, XC2267, XC2269, XC2272, XC2277, XC2278, XC2279, XC2280, XC2298, XC2858, XC3511, XC3916, XC4085* | -5.97-2.95 |
| (XI) Hypersensitive reaction and pathogenicity (19) | |  |
| Hrp related proteins | *XC3009, XC3010, XC3011, XC3012, XC3013, XC3014, XC3015, XC3019, XC3021, XC3025* | -2.75--1.51 |
| T3s effectors and virulence proteins | *XC0241, XC2004, XC2081, XC2602, XC2995, XC3160, XC3177, XC4273, XC2082* | -2.85--2.47 |
| (XII) Iron uptake (19) | *XC0124, XC0915, XC0925, XC1004, XC1165, XC1284, XC1341, XC1451, XC1644, XC2137, XC2194, XC2354, XC2355, XC2485, XC2512, XC2642, XC4044, XC4053, XC4146* | -4.34-1.70 |
| (XIII) Ribosomal proteins (12) | *XC0491, XC2670, XC3092, XC3093, XC3315, XC3321, XC3322, XC3324, XC3331, XC3337, XC3357, XC4122* | 1.41-2.19 |
| (XIV) Transcription regulators (27) | *XC0243, XC0245, XC0273, XC0556, XC0601, XC0689, XC0816, XC0832, XC0848, XC0891, XC1365, XC1745, XC1790, XC1909, XC2251, XC2639, XC2729, XC2840, XC2841, XC2934, XC3099, XC3175, XC4025, XC4055, XC4118, XC4142, XC4222* | -2.28-3.29 |
| (XV) Dehydrogenase (0) |  |  |
| (XVI) Aerobic and anaerobic respiration (17) | |  |
| Oxidases and oxidoreductase | *XC1597, XC2800, XC3138, XC3170, XC3762, XC3780* | -2.37-1.83 |
| Transferase | *XC0209, XC0882, XC0998, XC1000, XC1648, XC2321, XC2697, XC3951, XC4099, XC4230, XC4294* | -2.50-2.61 |
| (XVII) Membrane components and transporters (48) | *XC0017, XC0084, XC0155, XC0156, XC0167, XC0431, XC0432, XC0557, XC0600, XC0642, XC0744, XC0810, XC0811, XC1087, XC1142, XC1345, XC1346, XC1347, XC1459, XC1541, XC1619, XC1621, XC1647, XC1682, XC1840, XC1882, XC2728, XC2796, XC2846, XC2857, XC2859, XC2923, XC3320, XC3458, XC3576, XC3665, XC3788, XC3859, XC4014, XC4079, XC4094, XC4223, XC4224, XC4257, XC4291, XC4292, XC4293, XC4327* | -3.40-2.73 |
| (XVIII) Hypothetical proteins (245) | *XC0015, XC0022, XC0023, XC0030, XC0088, XC0089, XC0090, XC0105, XC0108, XC0168, XC0169, XC0177, XC0180, XC0230, XC0250, XC0252, XC0259, XC0260, XC0261, XC0262, XC0263, XC0266, XC0268, XC0288, XC0335, XC0343, XC0408, XC0426, XC0442, XC0464, XC0475, XC0526, XC0549, XC0563, XC0571, XC0606, XC0629, XC0653, XC0657, XC0720, XC0727, XC0771, XC0776, XC0817, XC0856, XC0877, XC0964, XC0971, XC0980, XC1019, XC1020, XC1044, XC1051, XC1068, XC1080, XC1083, XC1114, XC1126, XC1127, XC1137, XC1146, XC1147, XC1148, XC1156, XC1157, XC1174,*  *XC1179, XC1188, XC1215, XC1216, XC1281, XC1289, XC1294, XC1315, XC1321, XC1337, XC1339, XC1340, XC1351, XC1353, XC1371, XC1390, XC1401, XC1417, XC1453, XC1458, XC1460, XC1485, XC1493, XC1503, XC1527, XC1566, XC1581, XC1583, XC1646, XC1683, XC1709, XC1724, XC1732, XC1740, XC1807, XC1835, XC1979, XC1986, XC2042, XC2044, XC2088, XC2131, XC2132, XC2135, XC2144, XC2164, XC2187, XC2189, XC2190, XC2193, XC2200, XC2328, XC2353, XC2362, XC2415, XC2418, XC2419, XC2420, XC2423, XC2424, XC2437, XC2464, XC2479, XC2513, XC2539, XC2586, XC2587, XC2615, XC2631, XC2632, XC2634, XC2651, XC2773, XC2781, XC2808, XC2836, XC2860, XC2888, XC2898, XC2904, XC2922, XC2932, XC2945, XC2987, XC3074, XC3080, XC3100, XC3103, XC3107, XC3108, XC3109, XC3110, XC3111, XC3116, XC3119, XC3124, XC3150, XC3152, XC3171,*  *XC3174, XC3176, XC3189, XC3231, XC3253, XC3299, XC3372, XC3481, XC3483, XC3490, XC3523, XC3540, XC3543, XC3549, XC3553, XC3554, XC3555, XC3556, XC3562, XC3598, XC3645, XC3654, XC3690, XC3692, XC3693, XC3695, XC3696, XC3697, XC3715, XC3721, XC3722, XC3744, XC3746, XC3753, XC3781, XC3794, XC3834, XC3835, XC3851, XC3857, XC3862, XC3869, XC3870, XC3900, XC3903, XC3922, XC3940, XC3955, XC3956, XC3968, XC3969, XC3970, XC3977, XC3980, XC4024, XC4028, XC4029, XC4033, XC4039, XC4040, XC4047, XC4086, XC4116, XC4128, XC4129, XC4136, XC4140, XC4147, XC4148, XC4153, XC4188, XC4190, XC4206, XC4228, XC4229, XC4264, XC4268, XC4295, XC4312, XC4323* | -7.28-16.41 |
| (XIX) Environmental information processing (19) | *XC0496, XC1410, XC1582, XC1755, XC1841, XC1938, XC1939, XC1966, XC2163, XC2390, XC2457, XC2475, XC2506, XC2816, XC3117, XC3118, XC4031, XC4054, XC4150* | -3.55-2.09 |
| (XX ) Other proteins (9) | *XC0395, XC0665, XC1032, XC2087, XC2119, XC2122, XC2438, XC3034, XC3917* | -2.77-152 |
| Total gene number | *626* |  |

**Notes:** Ratio means the value of log2 ratio of RPKM (mutant / wild type).
